# Regional Variation in End-of-life Care Just Before Death Among the Oldest Old in Japan: A Descriptive Study

**DOI:** 10.2188/jea.JE20230364

**Published:** 2024-12-05

**Authors:** Michi Sakai, Naohiro Mitsutake, Tomohide Iwao, Genta Kato, Shuzo Nishimura, Takeo Nakayama

**Affiliations:** 1 Department of Health Informatics, Kyoto University School of Medicine and Public Health, Kyoto, Japan; 2 Comprehensive Unit for Health Economic Evidence Review and Decision Support (CHEERS), Research Organization of Science and Technology, Ritsumeikan University, Kyoto, Japan; 3 Department of Research, Institute for Health Economics and Policy (IHEP), Tokyo, Japan; 4 Institute for Advancement of Clinical and Translational Science (iACT), Kyoto University Hospital, Kyoto, Japan; 5 Solution Center for Health Insurance Claims, Kyoto University Hospital, Kyoto, Japan; 6 Kyoto University Graduate School, Kyoto, Japan

**Keywords:** the oldest old, health insurance claims, real-world data, end-of-life care

## Abstract

**Background:**

The use of life-sustaining treatment (LST) in the final stage of life is a major policy concern due to increased costs, while its intensity does not correlate with quality. Previous reports have shown declining trends in LST use in Japan. However, regional practice variations remain unclear. This study aims to describe regional variations in LST use before death among the oldest old in Japan.

**Methods:**

A descriptive study was conducted among patients aged 85 years or older who passed away between April 2013 and March 2014. The study utilized health insurance claims from Japan’s National Database (NDB) to examine the use of cardiopulmonary resuscitation (CPR), mechanical ventilation (MV), and admission to the acute care ward (ACW) in the last 7 days of life.

**Results:**

Among 224,391 patients, the proportion of patients receiving LST varied by region. CPR ranged from 8.6% (Chubu) to 12.9% (Shikoku), MV ranged from 7.1% (Chubu) to 12.3% (Shikoku), and admission to ACW ranged from 4.5% (Chubu) to 10.1% (Kyushu-Okinawa). The adjusted odds ratios (AOR) for regional variation compared with Kanto were as follows: CPR (in Shikoku, AOR 1.85; 95% confidence interval [CI], 1.73–1.98), MV (in Shikoku, AOR 1.75; 95% CI, 1.63–1.87), and ACW admission (in Kyushu-Okinawa, AOR 1.69; 95% CI, 1.52–1.88).

**Conclusion:**

The study presents descriptive information regarding regional differences in the utilization of LST for the oldest old in Japan. Further research is necessary to identify the factors that contribute to these variations and to address the challenge of improving the quality of end-of-life care.

## INTRODUCTION

Amidst global aging, the increase in the population of the oldest old, those aged 85 years or older,^1^ is particularly significant: this group is projected to increase by 239% from 2020 to 2050, reaching 6% in high-income countries by 2050.^2^ Japan, in particular, is experiencing rapid aging, with the proportion of the oldest old reaching 4.9% as of 2020 and projected to reach 9.2% by 2050.^3^ The mortality rate is expected to reach 16.4% in 2050, with approximately 1.59 million deaths, marking the arrival of a high-mortality society.^3^ The development of health care systems to address the growing demand for end-of-life care is a global challenge. The utilization of life-sustaining treatment (LST) for the elderly population in the final stages of life has raised significant policy concerns, as it increases medical costs, whereas care intensity does not necessarily indicate quality.^4^^–^^7^ It has become evident that the intensity of end-of-life care among the elderly has been decreasing over the years.^7^^–^^10^ We previously reported a decreasing trend in the use of LST before death, including cardiopulmonary resuscitation (CPR) and mechanical ventilation (MV), among the oldest old Japanese from 2012 to 2014.^7^

On the other hand, less is known about regional practice variations in LST before death among the oldest old. Previous research indicated regional variations in the limitations of LST^11^ and the use of intensive procedures among the elderly,^12^^–^^15^ but there is a lack of evidence focusing on the oldest old. The oldest old, due to multiple comorbidities^16^ and variations in disease trajectories leading to death,^17^ cannot be considered a homogeneous population compared to younger age groups.^4^ Data on the effectiveness of LST for the oldest old are insufficient, causing challenges in evidence-based decision-making and potentially leading to regional practice variations.^4^^,^^5^^,^^18^ It has been suggested that regional variations can be explained by modifiable supply-side factors^12^^,^^13^ such as access to home care nurses^12^ and hospice or palliative care^19^ and hospital bed availability.^20^ Elucidating regional practice variations may make it possible to reduce variations and contribute to the equalization of health care.^21^^,^^22^

In this study, we aimed to describe regional variations in LST just before death among the oldest old in Japan using the using nationally representative health insurance claims data of the patients deceased in the fiscal year 2013, which demonstrated a declining intensity.^7^

## METHODS

### Definition

The end-of-life is defined as a specific period of time before death, recorded in health insurance claims data. LSTs are defined as treatments that help prolong life without improving the medical condition, based on the American Medical Association Code of Medical Ethics.^23^

### Study design

A descriptive study was conducted to elucidate regional practice variations in LST in the last 7 days among the oldest old in Japan.

### Setting

This study used nationwide data of patients who died between April 2013 and March 2014 in the hospitals that provide health services covered by the national health insurance system in Japan.

### Participants

The eligibility criteria for patients were as follows: (1) died between April 2013 and March 2014; and (2) over 85 years of age at the time of death; and (3) had been admitted to a hospital in the last 7 days of life.

### Variables

The primary outcome was the utilization of LST and admission to the acute care ward (ACW) in the 7 days preceding death. We studied cardiopulmonary resuscitation (CPR) and mechanical ventilation (MV) as LST in accordance with our previous report.^7^ The Japanese health insurance claim codes CPR, MV, and ACW were used to identify each LST and ACW admission. CPR involves chest compressions, defibrillation, or tracheal intubation. MV can be either invasive positive pressure ventilation (IPPV) or noninvasive positive pressure ventilation (NPPV). ACW includes intensive care unit (ICU), high care unit (HCU), stroke care unit (SCU), or emergency admissions. Note that stays in the ICU, SCU, and emergency department that exceed 14 days, as well as stays in the HCU that exceed 21 days, are not eligible for reimbursement and therefore are not recorded in the National Database (NDB). As a result, it may not be possible to capture long-term ACW admissions prior to death.

The study evaluated the covariates of age, sex, and region of medical institution location. The hospitals where the patients died were identified at the prefecture level and divided into eight regions across Japan, from north to south: Hokkaido, Tohoku, Kanto, Chubu, Kinki, Chugoku, Shikoku, and Kyushu, in accordance with the previous nationwide survey on practice variations using NDB.^24^

### Data source

This study used nationwide inpatient health insurance claims from the NDB, which contains almost 100% digitized claims under universal health coverage and enables the analysis of care in nearly the entire oldest old age group. Data extracted from the NDB database by Ministry of Health, Labour and Welfare (MHLW) was received based on the following eligibility criteria: (1) age at death over 85 years; and (2) death occurring between April 2013 and March 2014. Deceased patients were identified based on the hospitalization outcomes recorded in the claims, which allowed us to identify in-hospital mortality from claims data with a high degree of accuracy.

### Statistical analysis

We calculated the proportion of decedents who received CPR, MV, and ACW admission in the last 7 days of life. To adjust for the impact of age and sex on practice variation, logistic regression analysis was conducted. The use of each LST and ASW admission was used as binary categorical response variables, while age and region were used as explanatory variables. Age was included as a categorical variable in 5-year age groups, since a continuous variable was not provided. The region was treated as a categorical variable, with Kanto having the highest population and number of patients used as the reference. We used SPSS version 29.0.1 (SPSS Inc., Chicago, IL, USA) for Windows for the analysis. We set the significance level of each test to 5%.

### Ethical consideration

The study was conducted according to the Ethical Guidelines for Medical and Biological Research Involving Human Subjects and guidelines for the use of NDB issued by the MHLW. The study protocol was approved by Kyoto University’s research ethics committee (R1558-1) and Ritsumeikan University’s Ethics Review Board for Medical Research Involving Human Subjects (2020-063). The Ethics Committee waived the requirement for informed consent, since NDB data are anonymized.

## RESULTS

**Figure 1.**
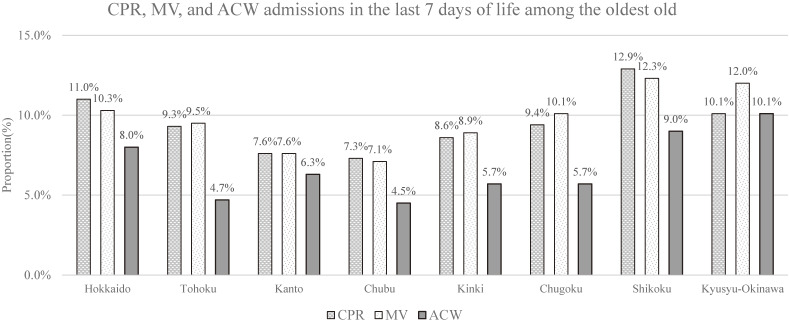
Administration of CPR, MV and ACW admissions in the last 7 days of life among the oldest old. ACW, acute care ward; CPR, cardiopulmonary resuscitation; MV, mechanical ventilation.

From April 2013 to March 2014, a total of 310,550 patients aged 85 years or older who died between April 2013 and March 2014 were identified. We excluded patients who had no records of hospitalization for 7 days or more prior to death (*n* = 77,153) and those with missing data or no record of treatment procedures before the date of death (*n* = 9,006), leaving 224,391 patients for analysis. The characteristics of the patients in each region were shown in Table 1. The proportion of patients receiving CPR ranged from 8.6% (in Chubu) to 12.9% (in Shikoku), MV ranged from 7.1% (in Chubu) to 12.3% (in Shikoku), and ACW admission ranged from 4.5% (in Chubu) to 10.1% (in Kyushu-Okinawa) (Table 2, Figure 1). The proportions for the 47 prefectures have been made available in the eTable 1. The adjusted odds ratios (AOR) for regional variation compared with Kanto were as follows: CPR (in Shikoku, AOR 1.85; 95% confidence interval [CI], 1.73–1.98), MV (in Shikoku, AOR 1.75; 95% CI, 1.63–1.87), and ACW admission (in Kyushu-Okinawa, AOR 1.69; 95% CI, 1.52–1.88) (Table 2).

**Table 1.** Characteristics of deceased patients

		Total	Hokkaido	Tohoku	Kanto	Chubu	Kinki	Chugoku	Shikoku	Kyusyu - Okinawa
	Number of Patients	224,391	12,549	19,862	58,589	38,191	37,856	16,988	9,497	30,859

Age, years	85–89	109,897 (49%)	5,956 (47.5%)	10,266 (51.7%)	29,163 (49.8%)	19,140 (50.1%)	18,590 (49.1%)	7,973 (46.9%)	4,453 (46.9%)	14,356 (46.5%)
	90–94	75,105 (33.5%)	4,274 (34.1%)	6,569 (33.1%)	19,578 (33.4%)	12,602 (33.0%)	12,542 (33.1%)	5,819 (34.3%)	3,187 (33.6%)	10,534 (34.1%)
	95–99	32,308 (14.4%)	1,906 (15.2%)	2,519 (12.7%)	8,168 (13.9%)	5,400 (14.1%)	5,487 (14.5%)	2,580 (15.2%)	1,460 (15.4%)	4,788 (15.5%)
	≥100	7,081 (3.2%)	413 (3.3%)	508 (2.6%)	1,680 (2.9%)	1,049 (2.7%)	1,237 (3.3%)	616 (3.6%)	397 (4.2%)	1,181 (3.8%)

sex	Male	90,016 (40.1%)	5,117 (40.8%)	7,832 (39.4%)	23,852 (40.7%)	16,041 (42.0%)	14,649 (38.7%)	6,837 (40.2%)	3,823 (40.3%)	11,865 (38.4%)
	Female	134,375 (59.9%)	7,432 (59.2%)	12,030 (60.6%)	34,737 (59.3%)	22,150 (58.0%)	23,207 (61.3%)	10,151 (59.8%)	5,674 (59.7%)	18,994 (61.6%)

Ward type	Acute	51,622 (23%)	10,327 (82.3%)	14,834 (74.7%)	42,177 (72.0%)	25,283 (66.2%)	27,138 (71.7%)	12,908 (76.0%)	7,478 (78.7%)	23,113 (74.9%)
	General	163,258 (72.8%)	1,819 (14.5%)	4,462 (22.5%)	13,934 (23.8%)	11,092 (29.0%)	9,150 (24.2%)	3,217 (18.9%)	1,608 (16.9%)	6,340 (20.5%)
	Long-term care	9,511 (4.2%)	403 (3.2%)	566 (2.8%)	2,478 (4.2%)	1,816 (4.8%)	1,568 (4.1%)	863 (5.1%)	411 (4.3%)	1,406 (4.6%)

**Table 2.** Administration of CPR, MV, and ACW admissions in the last 7 days of life among deceased patients

	Patients who received CPR, *n* (%) *n* = 224,391			Patients who received MV, *n* (%) *n* = 224,391			Patients who admitted to ACW, *n* (%) *n* = 51,622^a^		

	%	AOR^b^	95% CI	%	AOR	95% CI	%	AOR	95% CI
Total	8.8%			9.0%			6.2%		
Hokkaido	11.0%	1.52	(1.43–1.62)	10.3%	1.41	(1.32–1.51)	8.0%	1.29	(1.08–1.55)
Tohoku	9.3%	1.25	(1.18–1.32)	9.5%	1.27	(1.20–1.35)	4.7%	0.72	(0.62–0.84)
Kanto	7.6%	Ref		7.6%	Ref		6.3%	Ref	
Chubu	7.3%	0.96	(0.91–1.01)	7.1%	0.93	(0.88–0.97)	4.5%	0.70	(0.63–0.79)
Kinki	8.6%	1.15	(1.10–1.21)	8.9%	1.2	(1.14–1.26)	5.7%	0.90	(0.80–1.01)
Chugoku	9.4%	1.28	(1.21–1.36)	10.1%	1.39	(1.31–1.48)	5.7%	0.90	(0.77–1.06)
Shikoku	12.9%	1.85	(1.73–1.98)	12.3%	1.75	(1.63–1.87)	9.0%	1.49	(1.24–1.79)
Kyusyu–Okinawa	10.1%	1.40	(1.34–1.47)	12.0%	1.70	(1.62–1.78)	10.1%	1.69	(1.52–1.88)

ACW, acute care ward; AOR, adjusted odds ratio; CI, confidence interval; CPR, cardiopulmonary resuscitation; MV, mechanical ventilation.

^a^Only the patients admitted to institutions with ACW were included for ACW admission.

^b^Odds ratio adjusted for age and sex.

## DISCUSSION

The article describes the regional variations in the use of LST and ACW admission among the oldest old in Japan, using nationally representative health insurance claims data. Although the use of LST and ACW admission just before death has decreased,^7^ regional variations in practice were observed. The proportion was high in Shikoku, Kyushu-Okinawa, and Hokkaido, but low in Chubu.

The study’s findings provide descriptive insights into regional practice variation in end-of-life care for the oldest old. Therefore, direct comparison with previous studies is limited. However, our findings align with several reports on regional disparities in end-of-life care for adult patients.^11^^–^^15^ A multicenter study involving 36 countries across Asia, Europe, Central and South America, and Africa has shown significant global variations in the limitations of LST during the last 6 months of life among adult patients.^11^ The regional disparities in withholding treatment, withdrawing treatment, and failed CPR range from 20% (Africa) to 61% (Latin America) (with an average of 44%), 6 (Latin America) to 53% (Northern Europe) (average 36%), and 4 (Northern Europe) to 65% (Africa) (average 15%), respectively, while Asia (including China, Hong Kong, India, Japan, South Korea, Thailand) reported respective rates of 42%, 39%, and 17%. Research using claims databases also indicated regional variations in end-of-life care for the elderly within a single country.^12^^–^^15^ Among Medicare beneficiaries, there is more than a threefold difference in ICU admission rates during the last 2 years of life across regions (ranging from 7.6% to 22.0%).^14^ Surgical rates in the year preceding death also varied significantly by region, ranging from 11.5% to 34.4%.^15^ In Switzerland,^12^ the use of intensive procedures among patients aged ≥65 years in the last 6 months of life varied between regions, with differences in mechanical ventilation (MV) (2.4% to 4.8%) and ICU admission (10.8% to 14.2%).

There were significant limitations in explaining the factors associated with variation. We could not analyze the patient’s clinical background or facility characteristics, which may explain variations in the data included in the health insurance claims. There were also limitations in defining severity and cause of death from the recorded diagnosis and medical procedures. Furthermore, patient and family preferences, socioeconomic status, and family composition were not recorded. Due to privacy concerns, the results for regions with a low patient population cannot be disclosed, detailed examination of the characteristics of each area was not feasible. Due to the anonymization of facility names, a detailed examination of facility factors, such as the number of specialized medical staff, nurse staffing levels, and bed availability, was not possible. We could not cross-reference with other databases, which prevented us from assessing the utilization of nonmedical care services, such as caregiving services and hospice. Additionally, we focused on CPR, MV, and ACW admissions, so we did not comprehensively capture regional variations in end-of-life care. Furthermore, due to the low sensitivity for outpatient deaths,^25^ we utilized inpatient claims data, resulting in the exclusion of data in outpatient care. Nonetheless, our results could be useful for understanding end-of-life care, given that approximately 80% of Japanese people die in medical facilities.^26^ The utilization of NDB, which contains almost 100% of the digitized health insurance claims, allowed for the acquisition of reliable information on regional practice variation preceding death among the oldest old, despite the inherent limitations of descriptive studies.

The study presents descriptive information regarding regional differences in the utilization of LST for the oldest old. Further research is necessary to identify the factors that contribute to these variations and to address the challenge of improving the quality of end-of-life care.

## SUPPLEMENTARY MATERIAL

eTable 1. Use of CPR, MV, and ACW admissions in the last 7 days of life among the deceased patients^a^ by prefecture
