# Supplementary material for: Regional Variation in End-of-life Care Just Before Death Among the Oldest Old in Japan: A Descriptive Study
Source: J Epidemiol. 2024 Dec 5;34(12):600–4. doi: 10.2188/jea.JE20230364 (PMC11564063; doi:10.2188/jea.JE20230364)
Supplement: Supplementary file 1 [file je-34-600-s001.pdf]

eTable 1. Use of CPR, MV, and ACW admissions in the last 7 days of life among the deceased patients [a] by prefecture

	Use of CPR, MV			ACW Admission [b]		Population of the oldest old (in thousands, n) [d]	Proportion of the oldest old (%) [d]
	Total number of patients (n)	CPR Number of patients, (%)	MV Number of patients, (%)	Total number of patients (n)	Number of patients, (%)		
**Hokkaido**	12,549	1,377 (11.0%)	1,289 (10.3%)	1,819	145 (8.0%)	217	4.0%
**Tohoku**							
Aomori	2,446	309 (12.6%)	271 (11.1%)	337	21 (6.2%)	54	4.0%
Iwate	2,928	259 (8.8%)	286 (9.8%)	588	19 (3.2%)	60	4.6%
Miyagi	4,119	295 (7.2%)	219 (5.3%)	812	63 (7.8%)	84	3.6%
Akita	2,732	206 (7.5%)	247 (9.0%)	997	18 (1.8%)	55	5.2%
Yamagata	3,009	203 (6.7%)	250 (8.3%)	707	52 (7.4%)	60	5.3%
Fukushima	4,628	579 (12.5%)	621 (13.4%)	1,021	35 (3.4%)	89	4.6%
**Kanto**							
Ibaraki	5,644	604 (10.7%)	548 (9.7%)	981	68 (6.9%)	103	3.5%
Tochigi	3,364	217 (6.5%)	230 (6.8%)	700	41 (5.9%)	70	3.5%
Gunma	4,240	395 (9.3%)	431 (10.2%)	718	32 (4.5%)	77	3.9%
Saitama	10,135	773 (7.6%)	710 (7.0%)	1,974	88 (4.5%)	174	2.4%
Chiba	8,915	580 (6.5%)	517 (5.8%)	1,926	99 (5.1%)	171	2.8%
Tokyo	15,371	1,132 (7.4%)	1,215 (7.9%)	4,121	331 (8.0%)	375	2.8%
Kanagawa	10,920	727 (6.7%)	807 (7.4%)	3,514	219 (6.2%)	244	2.7%
**Chubu**							
Niigata	5,657	276 (4.9%)	265 (4.7%)	961	37 (3.9%)	113	4.8%
Yamanashi	2,455	230 (9.4%)	158 (6.4%)	757	21 (2.8%)	49	4.6%
Nagano	2,480	281 (11.3%)	236 (9.5%)	646	36 (5.6%)	48	4.1%
Toyama	1,922	226 (11.8%)	248 (12.9%)	404	20 (5.0%)	37	4.7%
Ishikawa	1,796	160 (8.9%)	159 (8.9%)	259	- (-) [c]	38	4.5%
Fukui	4,556	418 (9.2%)	406 (8.9%)	1,642	78 (4.8%)	107	5.0%
Gifu	3,622	265 (7.3%)	260 (7.2%)	1,329	48 (3.6%)	78	3.8%
Shizuoka	5,870	323 (5.5%)	348 (5.9%)	2,056	72 (3.5%)	140	3.8%
Aichi	9,833	607 (6.2%)	642 (6.5%)	3,038	182 (6.0%)	198	2.7%
**Kansai**							
Mie	2,867	304 (10.6%)	391 (13.6%)	908	20 (2.2%)	70	3.8%
Shiga	2,340	175 (7.5%)	170 (7.3%)	589	35 (5.9%)	46	3.2%
Kyoto	4,641	352 (7.6%)	405 (8.7%)	1,164	75 (6.4%)	94	3.6%
Osaka	13,804	1,182 (8.6%)	1,164 (8.4%)	3,107	206 (6.6%)	247	2.8%
Hyogo	9,475	827 (8.7%)	879 (9.3%)	2,216	136 (6.1%)	190	3.4%
Nara	2,472	178 (7.2%)	194 (7.8%)	627	15 (2.4%)	50	3.6%

Wakayama	2,257	219 (9.7%)	161 (7.1%)	539	32 (5.9%)	46	4.7%
**Chugoku**							
Tottori	1,374	93 (6.8%)	138 (10.0%)	410	- (-) [c]	30	5.2%
Shimane	2,014	108 (5.4%)	148 (7.3%)	462	28 (6.1%)	42	6.0%
Oakayama	4,114	418 (10.2%)	424 (10.3%)	775	65 (8.4%)	85	4.4%
Hiroshima	5,558	548 (9.9%)	576 (10.4%)	1,013	51 (5.0%)	117	4.1%
Yamaguchi	3,928	430 (10.9%)	431 (11.0%)	557	32 (5.7%)	70	4.9%
**Shikoku**							
Tokushima	1,747	238 (13.6%)	202 (11.6%)	267	29 (10.9%)	38	4.9%
Kagawa	2,255	297 (13.2%)	279 (12.4%)	490	40 (8.2%)	46	4.7%
Ehime	3,137	377 (12.0%)	356 (11.3%)	528	37 (7.0%)	69	4.9%
Kochi	2,358	317 (13.4%)	331 (14.0%)	323	39 (12.1%)	43	5.8%
**Kyushu-Okinawa**							
Fukuoka	9,348	825 (8.8%)	1,099 (11.8%)	2,184	307 (14.1%)	183	3.6%
Saga	2,073	273 (13.2%)	292 (14.1%)	271	16 (5.9%)	39	4.6%
Nagasaki	3,390	343 (10.1%)	401 (11.8%)	679	48 (7.1%)	67	4.8%
Kumamoto	4,335	403 (9.3%)	434 (10.0%)	754	89 (11.8%)	89	4.9%
Oita	2,821	352 (12.5%)	322 (11.4%)	532	21 (3.9%)	57	4.8%
Miyazaki	2,455	269 (11.0%)	311 (12.7%)	270	15 (5.6%)	52	4.6%
Kagoshima	4,727	543 (11.5%)	608 (12.9%)	822	67 (8.2%)	86	5.1%
Okinawa	1,710	122 (7.1%)	224 (13.1%)	828	76 (9.2%)	41	2.9%
Total	224,391	224,391 (8.8%)	20,303 (9.0%)	51,622	3,204 (6.2%)	4,539	3.6%

ACW, acute care ward; CPR, cardiopulmonary resuscitation; MV, mechanical ventilation.

[a] The deceased patients: 85 years of age or older

[b] Only patients admitted to institutions equipped with ACW were included for analysis.

[c] The number and percentage of cases in prefectures with fewer than 10 patients are not disclosed for the protection of personal information. The total subjects of analysis for ACW admission consist of the combined total of prefectures excluding Ishikawa and Tottori Prefecture.

[d] The population of the oldest old: (in thousands) and the proportion of the people aged ≥85 years as of October 2013 are shown as reference information.
